# 2-Isobutyl-6-phenyl­imidazo[2,1-*b*][1,3,4]thia­diazole

**DOI:** 10.1107/S1600536810053201

**Published:** 2011-01-08

**Authors:** Hoong-Kun Fun, Madhukar Hemamalini, D. Jagadeesh Prasad, Prakash Anil Castelino, V. V. Anitha

**Affiliations:** a X-ray Crystallography Unit, School of Physics, Universiti Sains Malaysia, 11800 USM, Penang, Malaysia; b Department of Chemistry, Mangalore University, Mangalore, Karnataka, India; c Department of Chemistry, St. Philomena’s College, Puttur, Dakshina Kannada, Karnataka, India

## Abstract

In the title compound, C_14_H_15_N_3_S, the imidazo[2,1-*b*][1,3,4]thia­diazole fused-ring system is close to planar, with a maximum deviation of 0.042 (1) Å, and the dihedral angle between it and the phenyl ring is 24.21 (6)°. The isobutyl group is disordered over two sets of sites in a 0.899 (9):0.101 (9) ratio. In the crystal, weak aromatic π–π stacking inter­actions involving the imidazole and thia­diazole rings with a centroid–centroid distance of 3.8067 (7) Å occur.

## Related literature

For applications of imidazo [2,1-*b*]-1,3,4-thia­diazole derivatives, see: Terzioglu & Gursoy (2003 ▶); Kolavi *et al.* (2006 ▶); Gadad *et al.* (2000 ▶); Andotra *et al.* (1997 ▶); Khazi *et al.* (1996 ▶); Andreani *et al.* (1982 ▶, 1987 ▶, 1991 ▶); Eberle & Robert (1977 ▶).
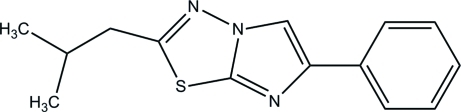


## Experimental

### 

#### Crystal data

C_14_H_15_N_3_S
*M* *_r_* = 257.35
Monoclinic, 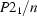

*a* = 5.6921 (1) Å
*b* = 19.6453 (4) Å
*c* = 12.3610 (2) Å
β = 96.127 (1)°
*V* = 1374.35 (4) Å^3^
*Z* = 4
Mo *K*α radiation
μ = 0.22 mm^−1^
*T* = 296 K
0.65 × 0.48 × 0.25 mm

#### Data collection

Bruker SMART APEXII CCD diffractometer
Absorption correction: multi-scan (*SADABS*; Bruker, 2009) ▶ *T*_min_ = 0.870, *T*_max_ = 0.947
36499 measured reflections
4038 independent reflections
3378 reflections with *I* > 2σ(*I*)
*R*_int_ = 0.028

#### Refinement

*R*[*F* ^2^ > 2σ(*F* ^2^)] = 0.039
*wR*(*F* ^2^) = 0.115
*S* = 1.03
4038 reflections
191 parameters
3 restraints
H-atom parameters constrained
Δρ_max_ = 0.31 e Å^−3^
Δρ_min_ = −0.30 e Å^−3^

### 

Data collection: *APEX2* (Bruker, 2009 ▶); cell refinement: *SAINT* (Bruker, 2009 ▶); data reduction: *SAINT*; program(s) used to solve structure: *SHELXTL* (Sheldrick, 2008 ▶); program(s) used to refine structure: *SHELXTL*; molecular graphics: *SHELXTL*; software used to prepare material for publication: *SHELXTL* and *PLATON* (Spek, 2009 ▶).

## Supplementary Material

Crystal structure: contains datablocks global, I. DOI: 10.1107/S1600536810053201/hb5776sup1.cif

Structure factors: contains datablocks I. DOI: 10.1107/S1600536810053201/hb5776Isup2.hkl

Additional supplementary materials:  crystallographic information; 3D view; checkCIF report

## Figures and Tables

**Fig. 1.**
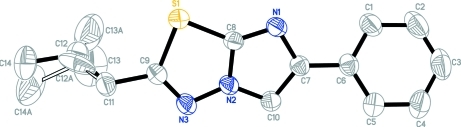
The asymmetric unit of the title compound, showing 50% probability displacement ellipsoids. Open bonds represent minor disorder components [H atoms are omitted for clarity].

**Fig. 2.**
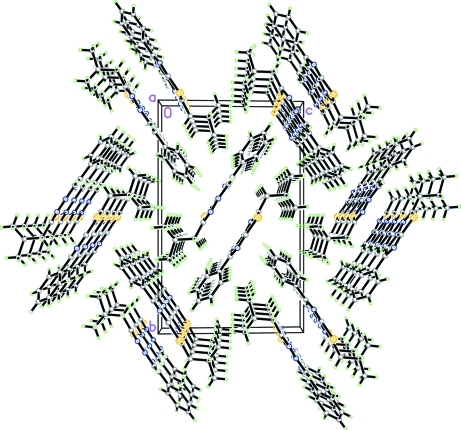
The crystal packing of the title compound (I).

## supplementary crystallographic information

### Comment

Imidazo [2,1-*b*]-1,3,4-thiadiazole derivatives are found to be biological active compounds possessing anticancer (Terzioglu & Gursoy, 2003), antitubercular (Kolavi *et al.*, 2006), antibacterial (Gadad *et al.*, 2000), antifungal (Andotra *et al.*, 1997), anticonvulsant, analgesic (Khazi *et al.*, 1996), anti-inflammatory (Andreani *et al.*, 1982), diuretic (Andreani *et al.*, 1991) and herbicidal activities (Andreani *et al.*, 1991). Moreover 1,3,4-thiadiazoles have many interesting biological activities, for example, 2-amino-5-(trifluoromethylphenyl alkyl)-1,3,4 thidadiazoles were used in the treatment of insomnia and anxiety (Eberle & Robert, 1977).

The title compound is shown in Fig. 1.  The imidazo[2,1-*b*] [1,3,4]thiadiazole (S1/N1–N3/C7–C10) ring is essentially planar, with a maximum deviation of  0.042 (1) Å for atom C7.  The isobutyl group is disordered over two sets of positions, with a refined occupancy ratio of 0.899 (9):0.101 (9). The dihedral angle between the imidazo[2,1-*b*] [1,3,4]thiadiazole (S1/N1–N3/C7–C10) ring and the benzene ring (C1–C6) is 24.21 (6)°.

In the crystal structure (Fig. 2), there are no classical hydrogen bonds and stabilization is achieved by weak  π–π stacking interactions between the thiadiazole (S1/N2–N3/C8–C9) ring and imidazole (N1–N2/C7–C8/C10) ring with centroid-to-centroid distance of 3.8067 (7) Å [symmetry code: 2-x, -y, 2-z].

### Experimental

5-isobutyl-1,3,4-thiadiazole-2-amine (1 molar equivalent) and phenacyl bromide (1 molar equivalent) are refluxed with ethanol for 4 hrs.  The solvent was then distilled off and the reaction mass was poured into the crushed ice. The resulting solid, 2-isobutylimidazo[2,1-*b*][1,3,4]thiadiazole, that separated out was filtered and dried.  The compound was recrystallized using ethanol and DMF mixture to form yellow blocks of the title compound. M.pt. 121–126°C.

### Refinement

All the H atoms were positioned geometrically [C–H = 0.93–0.98 Å] and were refined using a riding model, with *U*_iso_(H) = 1.2 or 1.5 *U*_eq_(C). The isobutyl group is disordered over two sites with refined occupancies of 0.899 (9):0.101 (9).

### Crystal data

**Table:**

C_14_H_15_N_3_S	*F*(000) = 544
*M**_r_* = 257.35	*D*_x_ = 1.244 Mg m^−^^3^
Monoclinic, *P*2_1_/*n*	Mo *K*α radiation, λ = 0.71073 Å
Hall symbol: -P 2yn	Cell parameters from 9875 reflections
*a* = 5.6921 (1) Å	θ = 2.7–30.3°
*b* = 19.6453 (4) Å	µ = 0.22 mm^−^^1^
*c* = 12.3610 (2) Å	*T* = 296 K
β = 96.127 (1)°	Block, yellow
*V* = 1374.35 (4) Å^3^	0.65 × 0.48 × 0.25 mm
*Z* = 4	

### Data collection

**Table:**

Bruker SMART APEXII CCD diffractometer	4038 independent reflections
Radiation source: fine-focus sealed tube	3378 reflections with *I* > 2σ(*I*)
graphite	*R*_int_ = 0.028
φ and ω scans	θ_max_ = 30.1°, θ_min_ = 2.0°
Absorption correction: multi-scan (*SADABS*; Bruker, 2009)	*h* = −8→8
*T*_min_ = 0.870, *T*_max_ = 0.947	*k* = −27→27
36499 measured reflections	*l* = −17→17

### Refinement

**Table:**

Refinement on *F*^2^	Primary atom site location: structure-invariant direct methods
Least-squares matrix: full	Secondary atom site location: difference Fourier map
*R*[*F*^2^ > 2σ(*F*^2^)] = 0.039	Hydrogen site location: inferred from neighbouring sites
*wR*(*F*^2^) = 0.115	H-atom parameters constrained
*S* = 1.03	*w* = 1/[σ^2^(*F*_o_^2^) + (0.0607*P*)^2^ + 0.2698*P*] where *P* = (*F*_o_^2^ + 2*F*_c_^2^)/3
4038 reflections	(Δ/σ)_max_ = 0.001
191 parameters	Δρ_max_ = 0.31 e Å^−^^3^
3 restraints	Δρ_min_ = −0.30 e Å^−^^3^

### Special details

**Table:**

Geometry. All esds (except the esd in the dihedral angle between two l.s. planes) are estimated using the full covariance matrix. The cell esds are taken into account individually in the estimation of esds in distances, angles and torsion angles; correlations between esds in cell parameters are only used when they are defined by crystal symmetry. An approximate (isotropic) treatment of cell esds is used for estimating esds involving l.s. planes.
Refinement. Refinement of F^2^ against ALL reflections. The weighted R-factor wR and goodness of fit S are based on F^2^, conventional R-factors R are based on F, with F set to zero for negative F^2^. The threshold expression of F^2^ > 2sigma(F^2^) is used only for calculating R-factors(gt) etc. and is not relevant to the choice of reflections for refinement. R-factors based on F^2^ are statistically about twice as large as those based on F, and R- factors based on ALL data will be even larger.

### Fractional atomic coordinates and isotropic or equivalent isotropic displacement parameters (Å^2^)

**Table:**

	*x*	*y*	*z*	*U*_iso_*/*U*_eq_	Occ. (<1)
S1	−0.10652 (5)	0.001167 (15)	0.83286 (3)	0.04296 (11)	
N1	−0.09710 (16)	0.12234 (5)	0.96090 (8)	0.0375 (2)	
N2	0.23227 (16)	0.07221 (5)	0.91884 (8)	0.0369 (2)	
N3	0.34801 (18)	0.02027 (5)	0.87202 (9)	0.0412 (2)	
C1	−0.1245 (2)	0.25685 (7)	1.06391 (14)	0.0544 (3)	
H1A	−0.2492	0.2450	1.0125	0.065*	
C2	−0.1425 (3)	0.31387 (9)	1.12942 (17)	0.0724 (5)	
H2A	−0.2792	0.3401	1.1213	0.087*	
C3	0.0404 (3)	0.33173 (9)	1.20606 (16)	0.0731 (5)	
H3A	0.0269	0.3697	1.2498	0.088*	
C4	0.2424 (3)	0.29331 (9)	1.21764 (14)	0.0688 (4)	
H4A	0.3664	0.3055	1.2691	0.083*	
C5	0.2629 (3)	0.23646 (8)	1.15317 (12)	0.0540 (3)	
H5A	0.4005	0.2106	1.1620	0.065*	
C6	0.0797 (2)	0.21756 (6)	1.07525 (10)	0.0395 (2)	
C7	0.10261 (19)	0.15721 (6)	1.00717 (9)	0.0361 (2)	
C8	−0.00847 (18)	0.07150 (6)	0.90932 (9)	0.0346 (2)	
C9	0.1913 (2)	−0.02035 (6)	0.82471 (10)	0.0387 (2)	
C10	0.3078 (2)	0.12707 (6)	0.98180 (10)	0.0406 (3)	
H10A	0.4625	0.1408	1.0026	0.049*	
C11	0.2544 (2)	−0.08474 (6)	0.77025 (11)	0.0456 (3)	
H11A	0.1916	−0.1229	0.8079	0.055*	0.899 (9)
H11B	0.4251	−0.0891	0.7778	0.055*	0.899 (9)
H11C	0.1818	−0.1211	0.8065	0.055*	0.101 (9)
H11D	0.4220	−0.0903	0.7866	0.055*	0.101 (9)
C12	0.1633 (7)	−0.08944 (18)	0.6491 (3)	0.0618 (9)	0.899 (9)
H12A	−0.0066	−0.0798	0.6399	0.074*	0.899 (9)
C13	0.2898 (12)	−0.03784 (19)	0.5858 (3)	0.1205 (16)	0.899 (9)
H13A	0.2567	0.0071	0.6105	0.181*	0.899 (9)
H13B	0.2362	−0.0418	0.5098	0.181*	0.899 (9)
H13C	0.4569	−0.0460	0.5970	0.181*	0.899 (9)
C14	0.2053 (8)	−0.16347 (16)	0.6126 (3)	0.0948 (11)	0.899 (9)
H14A	0.1100	−0.1940	0.6500	0.142*	0.899 (9)
H14B	0.3690	−0.1750	0.6295	0.142*	0.899 (9)
H14C	0.1629	−0.1673	0.5355	0.142*	0.899 (9)
C12A	0.205 (7)	−0.0960 (19)	0.666 (2)	0.081 (12)	0.101 (9)
H12B	0.0650	−0.1245	0.6708	0.097*	0.101 (9)
C13A	0.096 (9)	−0.0463 (17)	0.583 (3)	0.122 (14)	0.101 (9)
H13D	0.0313	−0.0705	0.5196	0.182*	0.101 (9)
H13E	0.2154	−0.0152	0.5641	0.182*	0.101 (9)
H13F	−0.0266	−0.0214	0.6131	0.182*	0.101 (9)
C14A	0.347 (8)	−0.147 (2)	0.601 (2)	0.119 (13)	0.101 (9)
H14D	0.2895	−0.1445	0.5251	0.179*	0.101 (9)
H14E	0.3267	−0.1923	0.6271	0.179*	0.101 (9)
H14F	0.5114	−0.1350	0.6105	0.179*	0.101 (9)

### Atomic displacement parameters (Å^2^)

**Table:**

	*U*^11^	*U*^22^	*U*^33^	*U*^12^	*U*^13^	*U*^23^
S1	0.03290 (16)	0.04292 (18)	0.0523 (2)	0.00072 (10)	0.00121 (12)	−0.00946 (12)
N1	0.0321 (4)	0.0376 (5)	0.0430 (5)	0.0029 (4)	0.0052 (4)	−0.0013 (4)
N2	0.0301 (4)	0.0383 (5)	0.0433 (5)	0.0006 (3)	0.0085 (3)	−0.0043 (4)
N3	0.0344 (5)	0.0421 (5)	0.0484 (6)	0.0028 (4)	0.0112 (4)	−0.0066 (4)
C1	0.0432 (7)	0.0453 (7)	0.0753 (9)	0.0023 (5)	0.0096 (6)	−0.0114 (6)
C2	0.0595 (9)	0.0530 (8)	0.1081 (14)	0.0077 (7)	0.0254 (9)	−0.0217 (9)
C3	0.0771 (11)	0.0602 (9)	0.0865 (12)	−0.0070 (8)	0.0290 (9)	−0.0330 (9)
C4	0.0735 (11)	0.0698 (10)	0.0628 (9)	−0.0057 (8)	0.0062 (8)	−0.0265 (8)
C5	0.0543 (8)	0.0548 (8)	0.0522 (7)	0.0028 (6)	0.0029 (6)	−0.0118 (6)
C6	0.0416 (6)	0.0358 (5)	0.0426 (6)	−0.0016 (4)	0.0116 (5)	−0.0015 (4)
C7	0.0354 (5)	0.0351 (5)	0.0383 (5)	−0.0004 (4)	0.0068 (4)	0.0000 (4)
C8	0.0297 (5)	0.0364 (5)	0.0378 (5)	0.0012 (4)	0.0038 (4)	0.0004 (4)
C9	0.0371 (5)	0.0393 (5)	0.0403 (6)	0.0034 (4)	0.0077 (4)	−0.0009 (4)
C10	0.0328 (5)	0.0413 (6)	0.0482 (6)	−0.0040 (4)	0.0071 (4)	−0.0070 (5)
C11	0.0472 (6)	0.0403 (6)	0.0503 (7)	0.0036 (5)	0.0107 (5)	−0.0065 (5)
C12	0.0621 (18)	0.0715 (19)	0.0501 (12)	0.0138 (12)	−0.0009 (11)	−0.0205 (13)
C13	0.182 (5)	0.123 (3)	0.0616 (15)	−0.005 (3)	0.037 (2)	0.0168 (16)
C14	0.104 (2)	0.0893 (19)	0.0898 (18)	0.0083 (16)	0.0063 (16)	−0.0506 (15)
C12A	0.049 (13)	0.082 (17)	0.11 (3)	0.033 (11)	−0.001 (12)	0.044 (17)
C13A	0.15 (4)	0.12 (3)	0.088 (19)	−0.01 (2)	0.00 (2)	−0.037 (19)
C14A	0.15 (3)	0.14 (3)	0.070 (14)	−0.02 (2)	0.020 (18)	−0.055 (16)

### Geometric parameters (Å, °)

**Table:**

S1—C8	1.7324 (11)	C11—C12	1.534 (3)
S1—C9	1.7605 (12)	C11—H11A	0.9700
N1—C8	1.3142 (14)	C11—H11B	0.9700
N1—C7	1.3963 (15)	C11—H11C	0.9600
N2—C8	1.3629 (14)	C11—H11D	0.9600
N2—C10	1.3714 (15)	C12—C13	1.509 (5)
N2—N3	1.3753 (13)	C12—C14	1.549 (4)
N3—C9	1.2895 (16)	C12—H12A	0.9800
C1—C6	1.3894 (18)	C13—H13A	0.9600
C1—C2	1.392 (2)	C13—H13B	0.9600
C1—H1A	0.9300	C13—H13C	0.9600
C2—C3	1.376 (3)	C14—H14A	0.9600
C2—H2A	0.9300	C14—H14B	0.9600
C3—C4	1.370 (3)	C14—H14C	0.9600
C3—H3A	0.9300	C12A—C13A	1.501 (18)
C4—C5	1.384 (2)	C12A—C14A	1.563 (18)
C4—H4A	0.9300	C12A—H12B	0.9800
C5—C6	1.3925 (19)	C13A—H13D	0.9600
C5—H5A	0.9300	C13A—H13E	0.9600
C6—C7	1.4678 (16)	C13A—H13F	0.9600
C7—C10	1.3753 (15)	C14A—H14D	0.9600
C9—C11	1.4946 (16)	C14A—H14E	0.9600
C10—H10A	0.9300	C14A—H14F	0.9600
C11—C12A	1.30 (3)		
			
C8—S1—C9	88.09 (5)	C12A—C11—H11C	106.0
C8—N1—C7	103.51 (9)	C9—C11—H11C	106.5
C8—N2—C10	107.97 (9)	C12—C11—H11C	107.0
C8—N2—N3	118.59 (9)	H11B—C11—H11C	111.4
C10—N2—N3	133.36 (10)	C12A—C11—H11D	107.0
C9—N3—N2	108.10 (9)	C9—C11—H11D	106.5
C6—C1—C2	120.06 (15)	C12—C11—H11D	115.0
C6—C1—H1A	120.0	H11A—C11—H11D	102.7
C2—C1—H1A	120.0	H11C—C11—H11D	106.5
C3—C2—C1	120.55 (16)	C13—C12—C11	109.6 (3)
C3—C2—H2A	119.7	C13—C12—C14	112.5 (3)
C1—C2—H2A	119.7	C11—C12—C14	107.1 (3)
C4—C3—C2	119.75 (15)	C13—C12—H12A	109.2
C4—C3—H3A	120.1	C11—C12—H12A	109.2
C2—C3—H3A	120.1	C14—C12—H12A	109.2
C3—C4—C5	120.38 (16)	C12—C13—H13A	109.5
C3—C4—H4A	119.8	C12—C13—H13B	109.5
C5—C4—H4A	119.8	H13A—C13—H13B	109.5
C4—C5—C6	120.69 (14)	C12—C13—H13C	109.5
C4—C5—H5A	119.7	H13A—C13—H13C	109.5
C6—C5—H5A	119.7	H13B—C13—H13C	109.5
C1—C6—C5	118.57 (12)	C12—C14—H14A	109.5
C1—C6—C7	121.03 (12)	C12—C14—H14B	109.5
C5—C6—C7	120.40 (11)	H14A—C14—H14B	109.5
C10—C7—N1	111.69 (10)	C12—C14—H14C	109.5
C10—C7—C6	127.46 (11)	H14A—C14—H14C	109.5
N1—C7—C6	120.84 (10)	H14B—C14—H14C	109.5
N1—C8—N2	112.63 (10)	C11—C12A—C13A	126 (2)
N1—C8—S1	138.83 (9)	C11—C12A—C14A	123 (2)
N2—C8—S1	108.52 (8)	C13A—C12A—C14A	105 (2)
N3—C9—C11	122.70 (11)	C11—C12A—H12B	97.6
N3—C9—S1	116.69 (9)	C13A—C12A—H12B	97.6
C11—C9—S1	120.53 (9)	C14A—C12A—H12B	97.6
N2—C10—C7	104.19 (10)	C12A—C13A—H13D	109.5
N2—C10—H10A	127.9	C12A—C13A—H13E	109.5
C7—C10—H10A	127.9	H13D—C13A—H13E	109.5
C12A—C11—C9	123.4 (13)	C12A—C13A—H13F	109.5
C9—C11—C12	114.71 (15)	H13D—C13A—H13F	109.5
C12A—C11—H11A	106.9	H13E—C13A—H13F	109.5
C9—C11—H11A	108.6	C12A—C14A—H14D	109.5
C12—C11—H11A	108.6	C12A—C14A—H14E	109.5
C12A—C11—H11B	100.8	H14D—C14A—H14E	109.5
C9—C11—H11B	108.6	C12A—C14A—H14F	109.5
C12—C11—H11B	108.6	H14D—C14A—H14F	109.5
H11A—C11—H11B	107.6	H14E—C14A—H14F	109.5
			
C8—N2—N3—C9	0.80 (15)	C9—S1—C8—N1	−177.43 (13)
C10—N2—N3—C9	177.01 (12)	C9—S1—C8—N2	1.10 (9)
C6—C1—C2—C3	−0.3 (3)	N2—N3—C9—C11	−176.63 (11)
C1—C2—C3—C4	0.3 (3)	N2—N3—C9—S1	0.17 (13)
C2—C3—C4—C5	−0.4 (3)	C8—S1—C9—N3	−0.77 (10)
C3—C4—C5—C6	0.3 (3)	C8—S1—C9—C11	176.10 (10)
C2—C1—C6—C5	0.2 (2)	C8—N2—C10—C7	−0.12 (13)
C2—C1—C6—C7	179.96 (14)	N3—N2—C10—C7	−176.62 (12)
C4—C5—C6—C1	−0.2 (2)	N1—C7—C10—N2	−0.26 (13)
C4—C5—C6—C7	−179.99 (13)	C6—C7—C10—N2	178.61 (11)
C8—N1—C7—C10	0.54 (13)	N3—C9—C11—C12A	−117 (2)
C8—N1—C7—C6	−178.42 (10)	S1—C9—C11—C12A	66 (2)
C1—C6—C7—C10	156.55 (13)	N3—C9—C11—C12	−121.5 (2)
C5—C6—C7—C10	−23.69 (19)	S1—C9—C11—C12	61.8 (2)
C1—C6—C7—N1	−24.67 (17)	C12A—C11—C12—C13	−89 (14)
C5—C6—C7—N1	155.09 (12)	C9—C11—C12—C13	67.7 (4)
C7—N1—C8—N2	−0.62 (13)	C12A—C11—C12—C14	33 (13)
C7—N1—C8—S1	177.88 (11)	C9—C11—C12—C14	−170.0 (2)
C10—N2—C8—N1	0.49 (13)	C9—C11—C12A—C13A	5(6)
N3—N2—C8—N1	177.59 (10)	C12—C11—C12A—C13A	30 (10)
C10—N2—C8—S1	−178.47 (8)	C9—C11—C12A—C14A	155 (3)
N3—N2—C8—S1	−1.36 (13)		

## References

[bb1] Andotra, C. S., Langer, T. & Kotha, A. (1997). *J. Indian Chem. Soc.* **74**, 125–127.

[bb2] Andreani, A., Bonazzi, D., Rambaldi, M., Fabbri, G. & Rainsford, K. D. (1982). *Eur. J. Med. Chem.* **17**, 271–274.

[bb3] Andreani, A., Rambaldi, M., Locatelli, A. & Andreani, F. (1991). *Collect. Czech. Chem. Commun.* **56**, 2436–2447.

[bb4] Andreani, A., Rambaldi, M., Mascellani, G. & Rugarli, P. (1987). *Eur. J. Med. Chem.* **22**, 19–22.

[bb5] Bruker (2009). *APEX2*, *SAINT* and *SADABS* Bruker AXS Inc., Madison, Wisconsin, USA.

[bb6] Eberle, M. K. & Robert, E. M. (1977). US Patent 4054665.

[bb7] Gadad, A. K., Mahajanshetti, C. S., Nimbalkar, S. & Raichurkar, A. (2000). *Eur. J. Med. Chem.* **35**, 853–857. 10.1016/s0223-5234(00)00166-5 11006486

[bb8] Khazi, I. A. M., Mahajanshetti, C. S., Gadad, A. K., Tamalli, A. D. & Sultanpur, C. M. (1996). *Arzneim. Forsch. Drug. Res.* **46**, 949–952. 8931885

[bb9] Kolavi, G., Hegde, V., Khan, I. & Gadad, P. (2006). *Bioorg. Med. Chem.* **14**, 3069–3080. 10.1016/j.bmc.2005.12.020 16406644

[bb10] Sheldrick, G. M. (2008). *Acta Cryst.* A**64**, 112–122. 10.1107/S0108767307043930 18156677

[bb11] Spek, A. L. (2009). *Acta Cryst.* D**65**, 148–155. 10.1107/S090744490804362X PMC2631630 19171970

[bb12] Terzioglu, N. & Gursoy, A. (2003). *Eur. J. Med. Chem.* **38**, 781–786. 10.1016/s0223-5234(03)00138-7 12932910

